# A Mad Doctor

**Published:** 1882-11-20

**Authors:** 


### *A MAD DOCTOR.*

It is stated that Dr. McLean, of Detroit, Mich., has instituted a libel suit against one of the city papers for $50,000. We don't know the provocation, but suppose the paper has been criticising the Doctor's professional conduct.

